# Crystallization–Foaming Coupling in Foam Glass-Ceramics from Multi-Source Coal Power Wastes

**DOI:** 10.3390/ma19132795

**Published:** 2026-07-01

**Authors:** Yan He, Boxiong Shen

**Affiliations:** 1 School of Chemical Engineering and Technology, Hebei University of Technology, Tianjin 300401, China; heyan187297@163.com; 2 Tianjin Key Laboratory of Clean Energy and Pollution Control, School of Energy and Environmental Engineering, Hebei University of Technology, Tianjin 300401, China

**Keywords:** coal fly ash, coal bottom ash, desulfurization gypsum, foam glass-ceramics, crystallization–foaming coupling

## Abstract

The large-scale disposal of coal fly ash (CFA), coal bottom ash (CBA), and desulfurization gypsum (DG) from coal-fired power plants poses serious environmental challenges, driving the need for high-value utilization strategies. In this study, we propose a synergistic approach to prepare foam glass-ceramics from CFA, CBA, and DG via a sintering-foaming method. The effects of sintering temperature (1200–1230 °C) and DG content (0–5 wt.%) on phase composition, pore structure, and overall material properties were systematically investigated. The optimal sample, obtained at 1220 °C with 2 wt.% DG exhibits outstanding comprehensive performance: a bulk density of 1.0030 g/cm^3^, porosity of 62.09%, compressive strength of 9.66 MPa, and thermal conductivity of 0.6156 W/(m·K). Additionally, it demonstrates excellent chemical stability, with acid resistance exceeding 96% and alkali resistance over 98%, while the leaching concentrations of heavy metals (Pb, Cr, Cu, Zn) remain far below regulatory limits. Mechanistic analysis reveals a crystallization–foaming coupling effect. At an appropriate DG content (2 wt.%), a synergy is established: bubble formation provides heterogeneous nucleation sites that promote crystal precipitation, while moderate crystallization increases melt viscosity and stabilizes the pore structure. Conversely, excessive DG (3–5 wt.%) reduces melt viscosity, leading to bubble coalescence and rupture, suppressed crystallization, and consequently deteriorated material properties. This work provides a theoretical foundation for the synergistic utilization of multiple power plant wastes and the structure–property regulation of foam glass-ceramics.

## 1. Introduction

With the continuous growth of global energy demand, coal is expected to remain a dominant component of China’s energy structure for the foreseeable future [1,2]. During coal-fired power generation, vast amounts of industrial solid wastes are produced, including coal fly ash (CFA), coal bottom ash (CBA), and desulfurization gypsum (DG). Although significant progress has been made in solid waste resource utilization in China in recent years, a considerable proportion of coal-fired power plant wastes is still disposed of via landfilling or stockpiling. These disposal methods not only occupy large areas of land but also pose potential environmental risks, such as heavy metal leaching, dust diffusion, and groundwater contamination [3,4,5]. Consequently, the high-value and large-scale utilization of coal-fired power plant solid waste has become a critical research priority in the field of solid waste resource management.

Foam glass-ceramics are functional materials that integrate the properties of glass and porous structures. Due to their high porosity, low thermal conductivity, high mechanical strength, excellent corrosion resistance, and superior chemical stability [6,7,8,9], they hold great promise for applications in building insulation, industrial thermal insulation, and sound-absorbing materials [10,11,12]. Compared with conventional foam glass, foam glass-ceramics can develop a certain proportion of crystalline phases during sintering, which significantly enhances the skeletal strength and thermal stability of the material. Therefore, tailoring the properties of foam glass-ceramics by regulating crystallization behavior and pore structure evolution has emerged as a key research direction.

In recent years, the preparation of foam glass-ceramics from industrial solid wastes has garnered growing research interest. Silica- and alumina-rich residues—such as coal fly ash (CFA), metallurgical slag, tailings, and waste glass—have been widely employed as raw materials to construct glass network structures [13,14,15,16,17]. For instance, Abba et al. [18] produced foam glass-ceramics from waste soda-lime glass and secondary aluminum slag via an alkali-activation coupled foaming process, achieving a compressive strength of 4.1 MPa, porosity of 86%, thermal conductivity of 0.125 W/(m·K), and bulk density of 0.284 g/cm^3^. Wang et al. [19] fabricated foam glass-ceramics using CFA, blast furnace slag, and desulfurization gypsum (DG) at 1200 °C, obtaining a bulk density of 1.21 g/cm^3^, a thermal conductivity of 0.153 W/(m·K), and a compressive strength of 11.23 MPa; the products also exhibited low heavy metal leaching toxicity, making them suitable for wall insulation applications. Similarly, Sarkar et al. [20] prepared foam glass-ceramics from red mud, CFA, and eggshell powder, achieving a total porosity of 33%, bulk density of 2.14 g/cm^3^, compressive strength of 109.75 MPa, and thermal conductivity of 0.93 W/(m·K), demonstrating their potential as load-bearing exterior wall tiles.

However, most existing studies have focused on the preparation of glass-ceramics using single solid waste systems or simple binary systems as raw materials, while investigations on the synergistic utilization of multiple coal-fired power plant solid wastes for glass-ceramics production remain relatively limited. CFA and CBA are rich in SiO_2_ and Al_2_O_3_, which can serve as the primary silica–alumina sources for the formation of foam glass-ceramics. DG contains a large amount of CaSO_4_, which can decompose at high temperatures to release gases such as SO_2_, thereby exhibiting potential foaming capability [13]. In addition, the CaO generated from DG decomposition can participate in the regulation of the glass network structure, significantly affecting melt viscosity and crystallization behavior [21]. Therefore, exploring the synergistic utilization of CFA, CBA, and DG for the preparation of foam glass-ceramics is not only beneficial for improving the resource utilization efficiency of solid wastes but also for achieving the synergistic regulation of material structure and properties.

Based on the above considerations, this study prepared foam glass-ceramics via a sintering-foaming process using CFA, CBA, and DG as the primary raw materials. The effects of sintering temperature and DG content on phase composition, pore structure evolution, and comprehensive material properties were systematically investigated, with a particular focus on the coupling mechanism between crystallization behavior and the foaming process. The main novelties of this work are threefold: (1) a multi-solid-waste synergistic utilization system based on CFA–CBA–DG was established, enabling the high-value utilization of typical coal-fired power plant wastes. (2) DG was employed simultaneously as a foaming gas source and a glass network modifier, and its synergistic effects on melt viscosity, crystallization behavior, and pore structure stability were elucidated. (3) The crystallization–foaming coupling mechanism during sintering was systematically revealed based on the interrelationships among new surface generation rate, porosity, and crystallinity, thereby providing a theoretical basis for the structural design and performance optimization of foam glass-ceramics derived from multiple solid wastes.

## 2. Materials and Experimental Procedures

### 2.1. Raw Materials

The CFA, CBA, and DG used in this study were collected from a coal-fired power plant located in Sanhe City, China. The CBA was melted at a high temperature of 1500 °C and then water-quenched to obtain metastable slag (MS). The chemical compositions of the three raw materials are listed in Table 1.

**Figure 1:**
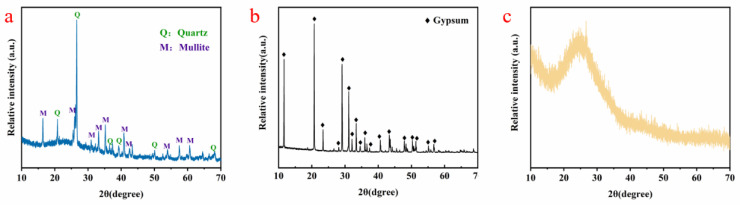
The XRD patterns of (**a**) CFA, (**b**) DG, and (**c**) MS.

The XRD patterns of the raw materials are presented in Figure 1. Quartz and mullite were identified as the major crystalline phases in CFA, while the primary crystalline phase in DG was CaSO_4_·2H_2_O. For the metastable slag (MS), only a broad and diffuse amorphous scattering hump was observed within the 2θ range of 10–70°, without any sharp characteristic diffraction peaks corresponding to crystalline phases. This result indicates that the metastable slag lacks a long-range ordered crystal structure and is predominantly composed of an amorphous phase, with no obvious crystal precipitation.

### 2.2. Sample Preparation

The mass ratio of CFA to MS was fixed at 4:6. DG was used as the foaming agent, with its addition ranging from 0 to 5 wt.%, and the resulting samples were designated as F0, F1, F2, F3, F4, and F5, respectively. To adjust the melt formation temperature and viscosity, 10 wt.% Na_2_B_4_O_7_·10H_2_O was added as a fluxing agent to promote liquid-phase formation and broaden the effective foaming temperature range. Additionally, 8 wt.% Na_3_PO_4_·12H_2_O was introduced as a foam stabilizer to enhance the viscoelasticity of the high-temperature melt, suppress bubble coalescence and rupture, and thereby facilitate the formation of a uniform and stable closed-pore structure.

**Figure 2:**
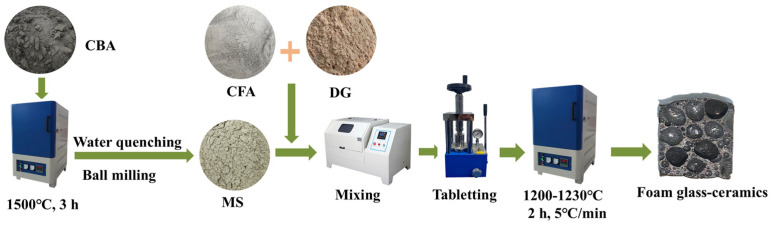
Preparation procedure of foam glass-ceramics.

All raw materials were accurately weighed according to the designed compositions and mixed by wet ball milling using anhydrous ethanol as the dispersing medium. After milling, the slurry was dried at 105 °C to constant weight. The dried powder was then uniaxially pressed into cylindrical specimens (20 mm in diameter and 5 mm in height) under a pressure of 10 MPa. The green bodies were further dried at 105 °C for 12 h to completely remove residual ethanol and adsorbed moisture. Subsequently, the samples were heated in a muffle furnace from room temperature to 1200–1230 °C at a rate of 5 °C/min, held for 2 h, and then naturally cooled to room temperature. Figure 2 shows the main experimental procedure.

### 2.3. Characterization Techniques

The chemical compositions of the raw materials were determined using X-ray fluorescence spectroscopy (XRF, XRF-1800, Shimadzu, Tokyo, Japan). The thermal behavior of DG under a nitrogen atmosphere was analyzed using thermogravimetric–differential scanning calorimetry (TG–DSC, SDT600, TA Instruments, New Castle, DE, USA) over the temperature range of 30–1400 °C at a heating rate of 10 °C/min.

The crystalline phases of the foam glass-ceramics were identified by X-ray diffraction (XRD, SMARTLAB SE, Rigaku, Tokyo, Japan). The XRD measurements were conducted at 40 kV and 30 mA, and the samples were scanned over a 2θ range of 10–70° at a scanning rate of 4°/min. The crystalline phases and their relative contents were determined by comparing the diffraction peaks with the standard patterns in the ICDD PDF-2 database (2004 edition). In addition, JADE software (v6) was used for peak fitting and area integration of the diffraction patterns, and the relative crystallinity was estimated based on the ratio of crystalline peak area to the total diffracted area (crystalline peaks plus amorphous background), providing a semi-quantitative evaluation of phase evolution.

The microstructures of the foam glass-ceramics were observed using field-emission scanning electron microscopy (SEM, Phenom Pure Plus, Phenom-World BV, Eindhoven, The Netherlands). The compressive strength of the samples was measured using an electronic universal testing machine (WDW-200, Jinan Zhongzheng Testing Machine Manufacturing, Jinan, China) at a crosshead speed of 2 mm/min. The thermal conductivity of the samples was measured using a thermal conductivity analyzer (TPS2500S, Hot Disk AB, Gothenburg, Sweden). Prior to testing, the samples were processed into blocks with identical dimensions and smooth surfaces to minimize measurement errors. Each sample was tested three times, and the average value was reported as the final result.

The bulk density of the samples was determined using the Archimedes method. Prior to measurement, the samples were dried and weighed to obtain the mass, denoted as m. A beaker filled with an appropriate amount of ultrapure water was placed on an electronic balance and tared. The sample was then suspended with a thin wire and slowly immersed in water until completely submerged without contacting the beaker wall or bottom. The balance reading was recorded as the sample volume (v). The bulk density was calculated according to the following equation:

(1) $$\rho =\frac{m}{v}$$

The samples were crushed and ground to below 100 mesh, and the true density (A) of the sample powders was measured using a pycnometer method. The porosity (P) of the samples was calculated according to the following equation:

(2) $$P=\left(1-\frac{A}{\rho }\right)\times 100\%$$

According to the Chinese Building Materials Industry Standard (JC/T 2097-2011 [22]), the acid and alkali resistance of the samples was evaluated using 20 wt.% H_2_SO_4_ solution and 20 wt.% NaOH solution, respectively, as follows:

(3) $$K=\frac{m}{m_{0}}\times 100\%$$

## 3. Results and Discussion

### 3.1. High-Temperature Thermal Behavior of DG

**Figure 3:**
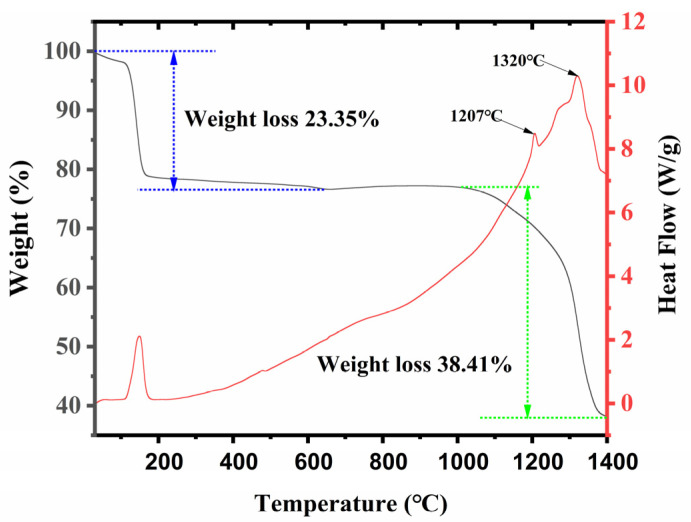
TG–DSC curves of DG.

To elucidate the thermal decomposition behavior of DG during the sintering of foam glass-ceramics, TG–DSC analysis was performed, and the results are presented in Figure 3. In the range from room temperature to 650 °C, the sample exhibited a mass loss of approximately 23.35%, accompanied by a distinct endothermic peak on the DSC curve. This indicates that the dehydration reaction of CaSO_4_·2H_2_O occurs predominantly in this stage, during which gypsum gradually transforms from dihydrate to hemihydrate and finally to anhydrous calcium sulfate.

Between 650 °C and 1000 °C, the TG curve remains relatively stable with only minor mass fluctuations, suggesting that the system enters a stable stage. At this point, DG has been largely converted to anhydrous CaSO_4_, whose crystal structure exhibits high thermal stability within this temperature range.

Above 1000 °C, the TG curve begins to decrease significantly, with a total mass loss reaching 38.41%. Simultaneously, a pronounced endothermic peak appears in the DSC curve, indicating the high-temperature decomposition of CaSO_4_ accompanied by the release of SO_2_ gas [23,24]. Notably, the decomposition temperature range is highly consistent with the softening temperature range of the glass matrix; therefore, it is inferred that the released gases can be effectively encapsulated by the highly viscous glassy liquid phase, thereby promoting the formation of a closed-pore structure.

### 3.2. Effect of Sintering Temperature

**Figure 4:**
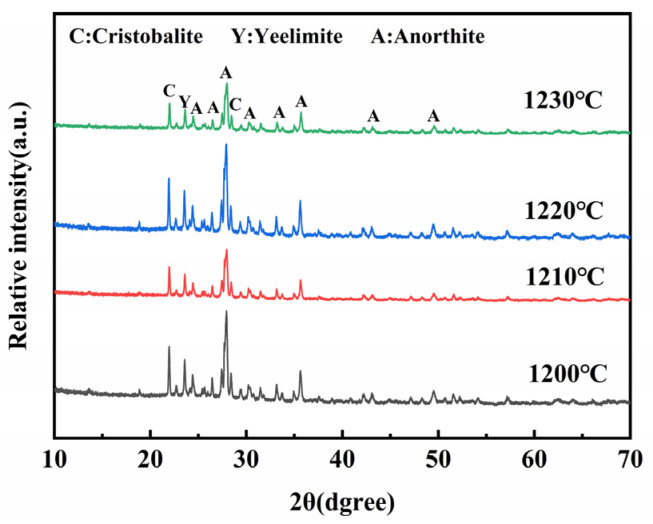
XRD patterns of foam glass-ceramics (F2) sintered at 1200–1230 °C.

Figure 4 shows the XRD patterns of foam glass-ceramics (F2) sintered at 1200–1230 °C. The results indicate that anorthite is the primary crystalline phase across this temperature range, along with minor amounts of tridymite and ettringite-type crystals, suggesting a relatively stable overall phase assemblage. The diffraction peak intensity does not vary strictly monotonically with increasing temperature. In particular, the sample treated at 1210 °C shows a relatively lower peak intensity compared with those at 1200 °C and 1220 °C, and is closer to that at 1230 °C. As the sintering temperature increases from 1200 °C to 1220 °C, the diffraction peak intensity of anorthite generally increases, reflecting enhanced crystallinity. This is attributed to the reduced viscosity of the liquid phase at higher temperatures, which facilitates ionic diffusion and thus accelerates nucleation and crystal growth. However, at 1230 °C, the diffraction intensities of some crystalline phases decrease, which implies that excessively low melt viscosity may promote partial remelting of the previously formed crystals.

**Figure 5:**
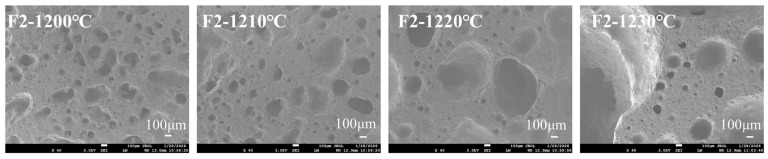
SEM images of foam glass-ceramics (F2) sintered at 1200–1230 °C.

Figure 5 shows SEM images of foam glass-ceramics (F2) sintered at 1200–1230 °C. At a sintering temperature of 1200 °C, only a limited number of small pores form within the sample. This is due to the relatively low foaming temperature, which leads to insufficient generation of the high-temperature liquid phase and a relatively high melt viscosity, incapable of effectively encapsulating the released gases. Consequently, most of the gas escapes through the interstitial voids between solid particles, resulting in poor foaming performance [25].

As the sintering temperature increases to 1220 °C, the pore structure becomes more uniform, with significantly larger pore sizes and improved sphericity. The pore walls remain continuous and intact. At this stage, the viscosity of the high-temperature liquid phase decreases to an appropriate range, and the decomposition of desulfurization gypsum proceeds sufficiently. The gas release rate becomes well matched with melt fluidity, facilitating stable bubble expansion under surface tension and effectively stabilizing bubbles within the glass matrix. This leads to a uniform distribution of well-developed closed pores.

When the sintering temperature is further raised to 1230 °C, the pore structure deteriorates markedly. As observed in micrographs, pore coalescence, a sharp increase in pore size, and irregular pore morphology occur, along with locally interconnected pores or collapsed structures. This is attributed to the excessively low melt viscosity at high temperature, which offers little resistance to bubble expansion. Excessive gas evolution, coupled with rapid bubble growth, thins the pore walls, making adjacent bubbles prone to coalescence into larger pores. In some cases, bubble rupture takes place, allowing gas to escape and resulting in pore collapse or the formation of open pores [26].

**Figure 6:**
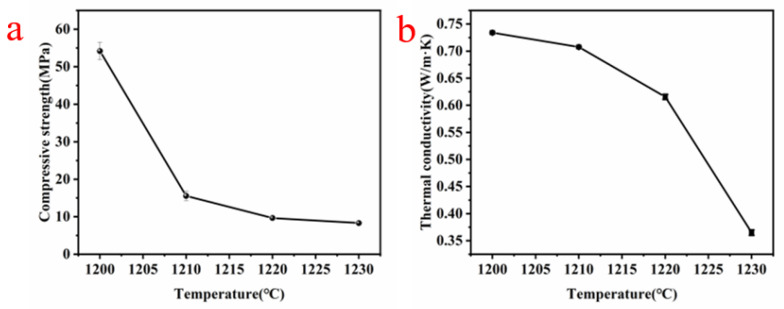
(**a**) Compressive strength and (**b**) thermal conductivity of F2 samples at different temperatures.

Figure 6 shows the compressive strength and thermal conductivity of F2 samples sintered at 1200–1230 °C. It can be observed that as the foaming temperature increases (>1200 °C), both the compressive strength and thermal conductivity of the foam glass-ceramics abruptly decrease. At 1200 °C, the system exhibits a relatively high melt viscosity and a slow gas release rate, resulting in only a small number of fine pores formed in the sample; thus, the compressive strength is relatively high. Meanwhile, due to the overall dense structure of the material, solid-phase conduction dominates heat transfer, leading to a relatively high thermal conductivity. As the foaming temperature rises, the viscosity of the glass phase decreases, and the gases released from the decomposition of the foaming agent can more easily expand within the melt to form a porous structure. The porosity of the material increases significantly, leading to a marked reduction in compressive strength. Furthermore, the thermal conductivity of the gas trapped in the pores is much lower than that of the solid framework, effectively weakening the heat transfer pathways and thereby gradually reducing the thermal conductivity [27].

### 3.3. Effect of DG Content

**Figure 7:**
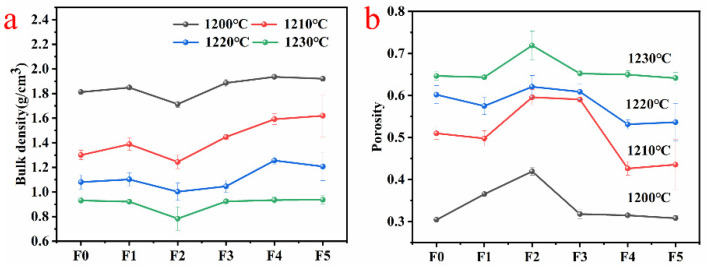
Effect of DG content on (**a**) bulk density and (**b**) porosity.

The effects of different foaming agent contents on the bulk density and porosity of foam glass-ceramics are shown in Figure 7. With increasing foaming agent content, the bulk density first decreases and then increases, while the porosity first increases and then decreases, indicating an inverse correlation between the two parameters.

**Figure 8:**
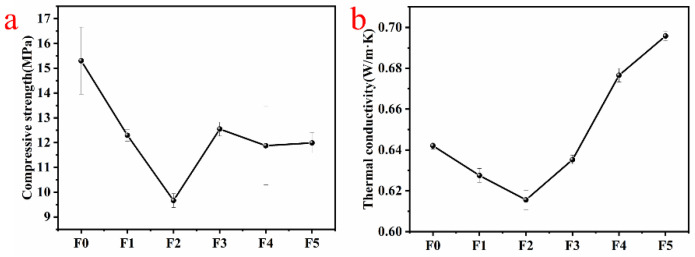
Effect of DG content on (**a**) compressive strength and (**b**) thermal conductivity.

**Figure 9:**
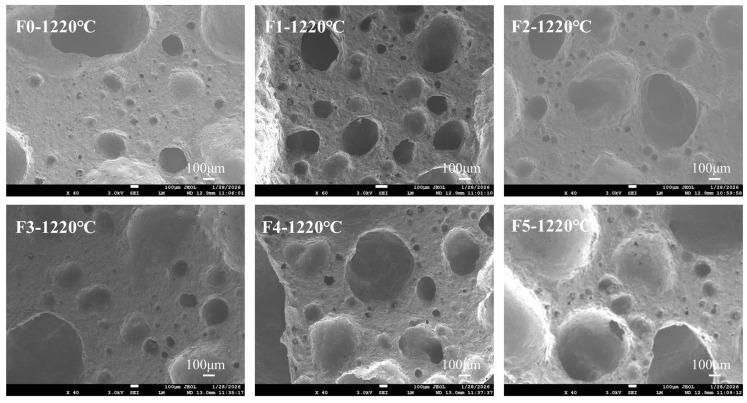
SEM images of foam glass-ceramics with different DG contents.

The effects of foaming agent content on the compressive strength and thermal conductivity of the foam glass-ceramics are presented in Figure 8. When the foaming agent content increases from 0% to 2%, DG decomposes at high temperatures, releasing gases that promote the formation of numerous uniformly distributed bubbles within the melt, as shown in Figure 9. Consequently, the porosity of the material gradually increases. Since the thermal conductivity of the gas phase is much lower than that of the dense glass matrix, the increased porosity significantly reduces the overall heat transfer capability of the material, leading to a gradual decrease in thermal conductivity. At the same time, the rise in porosity reduces the effective load-bearing area and weakens the skeletal structure, resulting in a corresponding decline in compressive strength [28].

Specifically, the significant decrease in compressive strength observed for sample F2 can be attributed to the most pronounced foaming state achieved at this stage. Combined with the porosity data and SEM observations, F2 exhibited the formation of a large number of uniformly distributed closed pores, which substantially reduced the effective load-bearing skeleton and intensified stress concentration effects, resulting in the lowest compressive strength value (9.66 MPa). Although a more uniform pore structure is beneficial for reducing thermal conductivity, the weakening effect of the highly porous framework became dominant, ultimately leading to the pronounced decline in compressive strength.

When the foaming agent content is further increased to 3–5%, excessive decomposition of DG generates a large amount of gas, which promotes bubble coalescence, rupture, and even escape. The originally uniform pore structure gradually transforms into large or interconnected pores, with some pores collapsing or being destroyed during the later stage of sintering. As a result, the overall porosity of the material decreases to some extent. Meanwhile, the enlarged and heterogeneous pore structure increases the proportion of the solid phase in the heat conduction pathways, leading to an increase in thermal conductivity.

**Figure 10:**
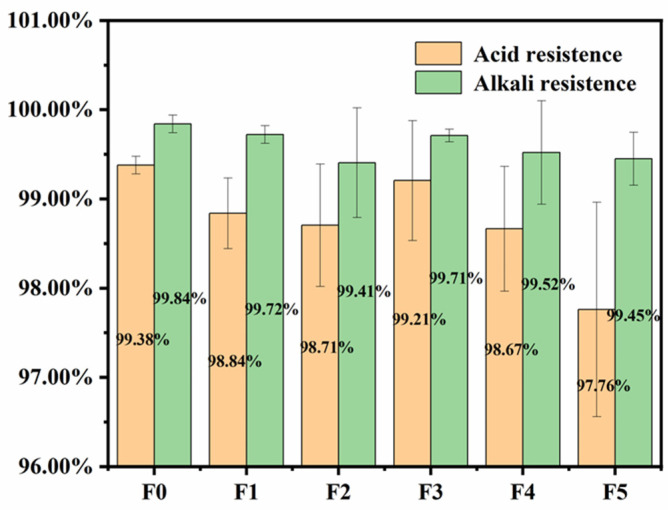
Acid resistance and alkali resistance of foam glass-ceramics prepared at 1220 °C.

Figure 10 illustrates the acid and alkali resistance of foam glass-ceramics prepared with varying DG contents at a foaming temperature of 1220 °C. With increasing foaming agent content, both acid and alkali resistance generally show a fluctuating downward trend. Nevertheless, the acid resistance of all samples remains above 96%, and the alkali resistance above 98%, indicating that the as-prepared foam glass-ceramics possess excellent chemical stability and satisfy the basic corrosion resistance requirements for industrial applications [29].

The Toxicity Characteristic Leaching Procedure (TCLP) was used to evaluate the leaching concentrations of typical heavy metals (Pb, Cr, Cu, Zn, and Cd) from the foam glass-ceramics prepared at 1220 °C; the results are presented in Table 2. Cd was not detected in any of the six samples sintered at this temperature. The leaching concentrations of Pb, Cr, Cu, and Zn were all far below the limits specified in the Chinese national standard (GB 5085.3-2007 [30]). These results demonstrate that the as-prepared foam glass-ceramics possess excellent heavy metal immobilization capability [31].

### 3.4. Interaction Mechanism Between Crystallization and Foaming

Surface tension and viscosity are fundamental physical properties of liquids (including melts) and are closely related to their internal structural characteristics. The surface tension of glass is defined as the work required to increase the interfacial area by one unit when the glass is in contact with another phase under constant temperature and constant volume conditions. For molten glass, the magnitude of surface tension directly determines whether bubbles can overcome the melt surface and escape. When the surface tension is relatively high, bubbles find it more difficult to escape; instead, they tend to accumulate and coalesce in the upper region of the melt, forming larger pores or cavities. In general, within a certain temperature range, the surface tension of glass exhibits an approximately linear relationship with temperature, which can be expressed by the following empirical equation [32]:

(4) $$A=A_{0}\left(1-b\Delta T\right)$$

where b is a constant related to the glass composition, A_0_ is the surface tension of the glass at the softening temperature T_0_ (N/m), and ΔT = T − T_0_ (°C).

Viscosity is a key parameter that describes the internal resistance of a fluid and characterizes its flow behavior. In the preparation of foam glass-ceramics, melt viscosity plays a decisive role in bubble formation and growth. At lower temperatures, the relatively high melt viscosity restricts gas diffusion and bubble expansion, resulting in slow bubble growth. Conversely, at excessively high temperatures, the melt viscosity decreases significantly, leading to rapid gas expansion and an increased likelihood of bubble rupture, which can result in open or interconnected pore structures. Previous studies have shown that the viscosity of glass melts decreases monotonically with increasing temperature. Moreover, the presence of fine crystalline particles and bubbles within the melt can also increase the viscosity to some extent [33]. In the preparation of foam glass-ceramics, the generation rate of new surface area (v) can be expressed by Equation (5) [34].

(5) $$v=\frac{A}{B}$$

In this equation, A represents the surface tension, and B represents the viscosity.

Meanwhile, the relationship between the new surface generation rate (v) and foaming time (t) can be described as follows [34]:

(6) $$t=\frac{\sqrt[{3}]{P}}{60v}\times \frac{\rho_{f}}{\rho_{b}}$$

In this expression, t is the foaming time, P is the porosity, ρf is the true density of the foam glass-ceramics, and ρb is the bulk density of the foam glass-ceramics.

Based on the experimentally measured data, including bulk density, true density, and porosity of the foam glass-ceramics, the new surface generation rate (v) was calculated using Equation (6), and the results are listed in Table 3.

**Figure 11:**
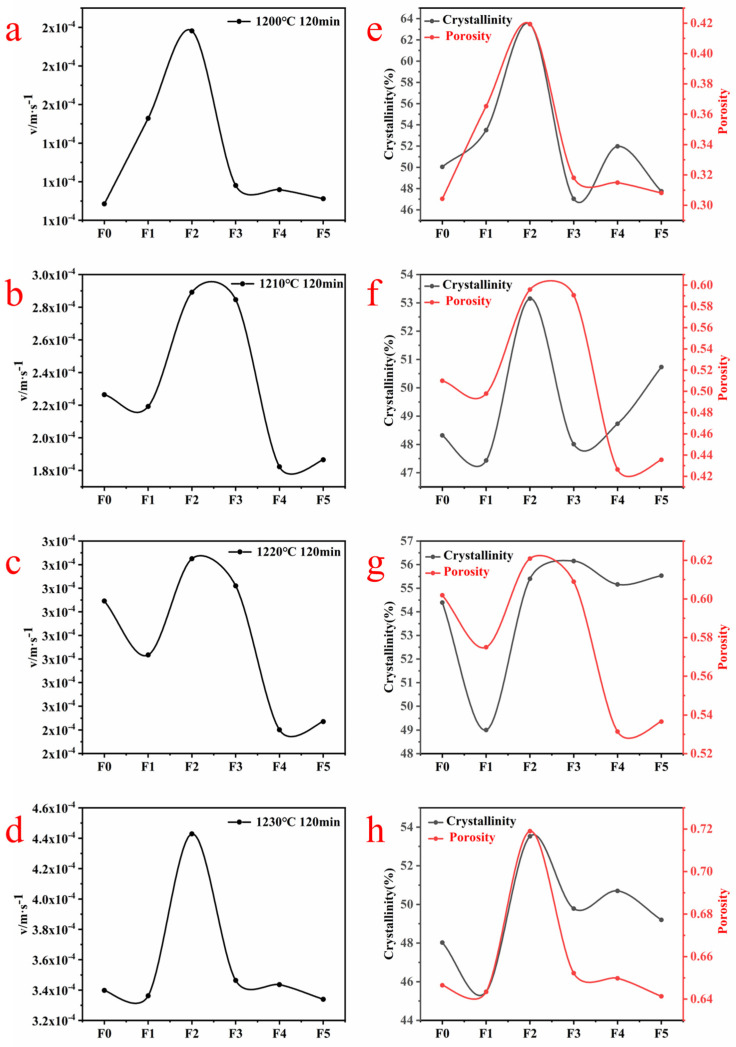
v values (**a**–**d**), porosity (**e**–**h**), and crystallinity of foam glass-ceramics with different foaming agent contents.

Figure 11 illustrates the variations in the new surface generation rate (v), porosity, and crystallinity of foam glass-ceramics with different foaming agent contents. Overall, as the foaming agent content increases, all three parameters first increase and then decrease, reaching their maxima for sample F2. This trend indicates a strong coupling between crystallization behavior and the foaming process during sintering, both of which jointly influence pore structure evolution and the final material properties [35].

As shown in Figure 11a–d, at sintering temperatures of 1200–1230 °C, the new surface generation rate (v) increases significantly when the foaming agent content rises from 0% to 2%, peaking at F2. At low foaming agent contents (F0–F1), the amount of gas generated is limited, resulting in fewer bubbles and fewer newly formed internal interfaces; consequently, the v value remains low.

When the foaming agent content reaches 2%, DG decomposes more effectively at high temperatures, releasing gases such as SO_2_, which leads to a substantial increase in bubble number and expansion. This creates more internal pore interfaces, markedly increasing v. At the same time, the formation of an appropriate number of bubbles promotes local structural rearrangement of the melt and provides additional heterogeneous nucleation sites for crystal growth, thereby simultaneously increasing crystallinity.

Figure 11e–h further illustrates the relationship between porosity and crystallinity as a function of foaming agent content. When the DG content increases from 0% to 2%, both porosity and crystallinity rise concurrently, indicating that moderate bubble growth promotes crystalline phase precipitation during crystallization. Conversely, appropriate crystallization increases melt viscosity and enhances pore wall mechanical strength, thereby stabilizing bubble expansion and facilitating a more uniform pore structure. Thus, at an optimal foaming agent content, a synergistic effect emerges between foaming and crystallization, where each process mutually reinforces the other.

When the foaming agent content is further increased to 3–5%, the excessive generation of gas from DG decomposition accelerates bubble formation. This leads to increased bubble coalescence, rupture, and escape, reducing the number of effective pore structures and lowering v. Moreover, excess Ca^2+^ released from DG enters the glass network and competes with Si^4+^ for O^2−^, breaking Si–O–Si bonds and reducing melt viscosity [19,36]. The lower viscosity further promotes rapid bubble expansion and escape, intensifying coalescence and rupture. In addition, the rapid release of large amounts of gas destabilizes the melt structure and weakens crystal nucleation and growth, resulting in decreased crystallinity. At the same time, excessive expansion may thin or even collapse pore walls, preventing stable preservation of some pores and ultimately reducing porosity.

Furthermore, as the sintering temperature increases from 1200 °C to 1230 °C, both v and porosity show a gradual upward trend, attributed to the reduced melt viscosity at higher temperatures. This facilitates bubble growth, thereby promoting the formation of new interfaces and the precipitation of crystalline phases.

## 4. Conclusions

(1) Foam glass-ceramics were successfully fabricated via a sintering-foaming method using CFA, CBA, and DG as raw materials. With increasing sintering temperature, the bulk density, compressive strength, and thermal conductivity of the foam glass-ceramics decreased, while the porosity increased. The pore structure gradually coarsened, and the pore size became more uniform. When the sintering temperature further increased to 1230 °C, pore coalescence occurred, leading to the formation of irregular, large pores. As the DG content increased from 0% to 5%, the bulk density, compressive strength, and thermal conductivity of the foam glass-ceramics first decreased and then increased, whereas the porosity showed an opposite trend. The sample containing 2% DG sintered at 1220 °C exhibited the best overall performance, with a bulk density of 1.0030 g/cm^3^, porosity of 62.09%, compressive strength of 9.66 MPa, and thermal conductivity of 0.6156 W/m·K. The samples also showed excellent chemical durability, with acid resistance ≥ 96% and alkali resistance ≥ 98%. The leaching concentrations of heavy metals (Pb, Cr, Cu, Zn, and Cd) were far below the regulatory limits, indicating their potential applicability in building thermal insulation materials.

(2) The coupling effect between crystallization and foaming during the sintering process of foam glass-ceramics was investigated. At an optimal DG content of 2%, moderate foaming increased heterogeneous nucleation sites and promoted crystal precipitation, while appropriate crystallization enhanced melt viscosity and stabilized the pore structure, resulting in a synergistic effect. However, when the DG content ranged from 3% to 5%, rapid gas release disrupted the melt structure, inhibited crystallization, and deteriorated the pore architecture, leading to a simultaneous decline in material performance.

(3) This study demonstrates the high-value utilization of multiple coal-fired power plant solid wastes.

## Figures and Tables

**Table 1:** Chemical compositions of CBA, CFA, and DG (wt.%).

	SiO_2_	Al_2_O_3_	CaO	Fe_2_O_3_	TiO_2_	K_2_O	SO_3_	Na_2_O	MgO	P_2_O_5_
BFA	47.39	28.184	11.448	5.939	1.795	1.637	1.417	0.735	0.491	0.376
CFA	48.65	28.433	10.808	6.459	1.781	1.519	0.915	0.457	0.383	0.165
DG	2.429	0.553	45.669	0.33	-	0.185	49.623	-	1.103	0.022

**Table 2:** Heavy metal leaching concentrations of foam glass-ceramics prepared at 1220 °C (mg/L).

Sample	Heavy Metal Leaching Concentration				
	Pb	Cr	Cu	Zn	Cd
F0-1220°C	0.004	0.101	0.036	0.110	ND
F1-1220°C	0.004	0.096	0.038	0.102	ND
F2-1220°C	0.003	0.041	0.041	0.069	ND
F3-1220°C	0.005	0.067	0.047	0.077	ND
F4-1220°C	0.006	0.099	0.042	0.075	ND
F5-1220°C	0.008	0.156	0.057	0.102	ND
Toxicity threshold	5.0	15.0	100	100	1.0

Note: ND indicates not detected.

**Table 3:** The new surface generation rate values of foam glass-ceramics with different foaming agent contents.

Sample	Bulk Density (g/cm^3^)	True Density (g/cm^3^)	Porosity	V (m·s^−1^)
F0-1200°C	1.8124	2.6049	0.3043	1.3425 × 10^−4^
F1-1200°C	1.8489	2.9130	0.3653	1.5643 × 10^−4^
F2-1200°C	1.7140	2.9522	0.4194	1.7907 × 10^−4^
F3-1200°C	1.8864	2.7663	0.3181	1.3903 × 10^−4^
F4-1200°C	1.9358	2.8257	0.3149	1.3793 × 10^−4^
F5-1200°C	1.9206	2.7758	0.3081	1.3558 × 10^−4^
F0-1210°C	1.3005	2.6536	0.5099	2.2641 × 10^−4^
F1-1210°C	1.3886	2.7650	0.4978	2.1918 × 10^−4^
F2-1210°C	1.2439	3.0774	0.5958	2.8913 × 10^−4^
F3-1210°C	1.4474	3.5348	0.5905	2.8457 × 10^−4^
F4-1210°C	1.5922	2.7757	0.4264	1.8224 × 10^−4^
F5-1210°C	1.6200	2.8699	0.4355	1.8605 × 10^−4^
F0-1220°C	1.0812	2.7159	0.6019	2.9457 × 10^−4^
F1-1220°C	1.1029	2.5948	0.5750	2.7170 × 10^−4^
F2-1220°C	1.0030	2.6454	0.6209	3.1251 × 10^−4^
F3-1220°C	1.0461	2.6746	0.6089	3.0098 × 10^−4^
F4-1220°C	1.2561	2.6804	0.5314	2.4006 × 10^−4^
F5-1220°C	1.2073	2.6052	0.5366	2.4354 × 10^−4^
F0-1230°C	0.9312	2.6345	0.6465	3.3977 × 10^−4^
F1-1230°C	0.9216	2.5843	0.6434	3.3620 × 10^−4^
F2-1230°C	0.7837	2.7893	0.7190	4.4285 × 10^−4^
F3-1230°C	0.9242	2.6575	0.6523	3.4634 × 10^−4^
F4-1230°C	0.9348	2.6697	0.6499	3.4356 × 10^−4^
F5-1230°C	0.9379	2.6149	0.6413	3.3393 × 10^−4^

## Data Availability

The original contributions presented in this study are included in the article. Further inquiries can be directed to the corresponding author.
